# Determination of the Thickness of Nanometer-Thick β‑Ga_2_O_3_ Membranes from Optical Interference and Colorimetric Analysis for Applications in Next-Generation Semiconductors

**DOI:** 10.1021/acsanm.6c02052

**Published:** 2026-06-27

**Authors:** Onur Çakıroğlu, Paula Pérez-Peinado, Emilio Nogales, Bianchi Méndez

**Affiliations:** Departamento de Física de Materiales, Facultad de Ciencias Físicas, 213131 Universidad Complutense de Madrid, 28040 Madrid, Spain

**Keywords:** wide-bandgap semiconductor, gallium oxide, nanomembranes, optical characterization

## Abstract

β-phase gallium oxide is emerging as a next-generation semiconductor material due to its ultrawide bandgap, high breakdown voltage, capabilities as a solar-blind ultraviolet photodetector, and resistance to extreme conditions. β-Ga_2_O_3_ membranes are available in various thicknesses and can be characterized by using standard techniques, e.g., atomic force microscopy (AFM). In this work, the use of optical interference patterns is demonstrated as an alternative characterization technique, highlighting its noncontact, user-friendly, and high-throughput thickness assessment for submicron-thick β-Ga_2_O_3_ membranes. Thicknesses of (100) β-Ga_2_O_3_ membranes on 290 nm SiO_2_/Si substrates are determined by applying Fresnel’s law to fit the contrast profile via optical interference patterns, while accuracy is assessed by atomic force microscopy. Moreover, the color map, calculated to assign the observed color of the membranes to their thickness, shows excellent agreement with the experimental results. The color-to-thickness correspondence is not always univocal and shows repeating color trends over thickness. Membranes of lesser thickness display colors of red, green, and pink, whereas membranes exceeding 600 nm predominantly exhibit orange and yellow tones. Finally, total color differences (TCD) analysis illustrates that (100) β-Ga_2_O_3_ membranes on 129, 221, 310, 392, and 467 nm SiO_2_/Si substrates have the best contrast; thus, membranes are more distinguishable.

## Graphical abstract

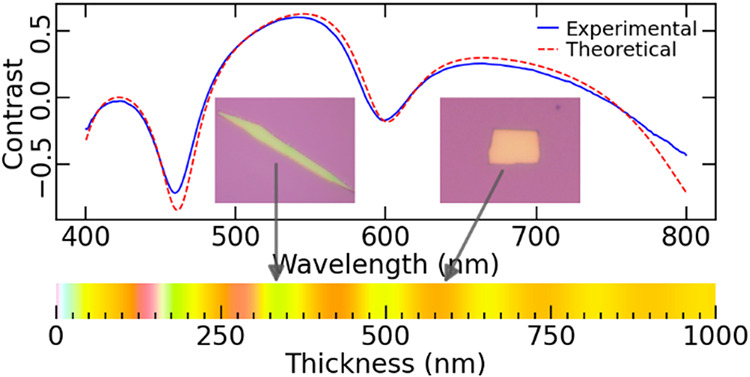

## 1 Introduction

Gallium oxide (Ga_2_O_3_) is leading the dawn of power electronics based on ultrawide-bandgap semiconductors (∼4.7 eV), particularly due to its stable β phase (β-Ga_2_O_3_), which exhibits remarkable optical and electronic properties.^1–4^ It has been proposed as a candidate for solar-blind ultraviolet (UV) photodetectors because of its outstanding optical properties, particularly in the 200–280 nm wavelength range.^5–7^ Furthermore, its stability under high voltage makes it potentially a critical component in power electronics.^8–11^ Nanostructured gallium oxide has also been analyzed in different shapes, including nanowires and nanoparticles.^12,13^ Meanwhile, its membrane form is anticipated to be advantageous due to its wide aspect ratio, which can also be further reduced in thickness through mechanical exfoliation techniques, in order to be integrated into van der Waals heterostructures.

The thickness of Ga_2_O_3_ membranes can determine their properties like changing their mechanical flexibility, optical absorption, or electrical resistance, similar to other thin metal oxides.^14–16^ Moreover, the thickness of various materials can be quantitatively determined by atomic force microscopy (AFM) or transmission/scanning electron microscopy (TEM/SEM) from their section.^17–19^ An optical method proposed around 2000s for two-dimensional materials has been successfully adapted for characterizing oxide materials. This optical method takes advantage of the interference pattern of reflectance across all media, given that all required parameters, except the thickness of the materials, are known.^20–23^ To date, no research has been specifically documented regarding Ga_2_O_3_.

Therefore, in this paper, we discuss the relation between color and thickness of (100) β-Ga_2_O_3_ membranes and report repeating color trends over thickness experimentally. Different thicknesses of Ga_2_O_3_ membranes produce various colors due to optical interference. Later, the applicability of Fresnel’s law with reflectance contrast spectra for Ga_2_O_3_ in determining thickness precisely will be given, showing accuracy via atomic force microscopy. Moreover, by using colorimetric analysis and creating a color bar, a high-throughput and noncontact method will be discussed to determine the thickness of submicron Ga_2_O_3_ membranes. Lastly, total color difference (TCD) analysis will be given to show which thickness values of the oxide layer of SiO_2_/Si substrates result in the best contrast of Ga_2_O_3_ membranes to observe them better.

## 2 Experiments and Methods

### 2.1 Sample Preparation

Commercial Novel Crystal Technology (100) bulk, unintentionally doped β-Ga_2_O_3_ crystals were mechanically exfoliated. The bulk crystals are partially attached to PVC blue adhesive tape (Nitto SPV224), and subsequently, the PVC tape is repeatedly folded over and then removed to obtain thinner crystals. Then, Ga_2_O_3_ membranes are mechanically exfoliated on the commercial (Graphene Supermarket) 290 nm SiO_2_/Si substrate from the adhesive tape. The tape is employed multiple times for exfoliation without undergoing preparation prior to each exfoliation from the primary bulk crystal.

### 2.2 Micro-Reflectance Spectroscopy

We acquired the reflectance spectra using a setup similar to that described in the article by Frisenda et al.^24^ The projected fiber core size is 12.7 μm as lateral resolution in this study. During acquisition, the microscope field diaphragm is reduced to its minimum size (115 μm) to minimize any influence from regions outside the target area.

### 2.3 Thickness Determination via AFM

The membrane thicknesses were measured using AFM (Nanotec Electronics) in contact mode. A prior step involved applying a background correction procedure to all AFM topography images before performing the thickness analysis. This was essential to eliminate the inherent tilt and bow artifacts caused by the AFM’s piezoelectric scanner’s nonlinear response, which appear as a polynomial background and can substantially make thickness determination harder.^25^ The membrane thicknesses were determined using the profile graph from the corrected topography images, which involved fitting a step function in Gwyddion software.

### 2.4 Reflectance Intensity via Fresnel’s Law

The number of media originating from each surface alters the reflectance intensity formulas. The physical system of air/SiO_2_/Si substrate is composed of three distinct media, whereas, when the Ga_2_O_3_ membrane is added on the substrate, it consists of four different media. Hence, the formulas are expressed for *I*_subs_ and *I*_memb_ as follows

1 $$I_{\mathrm{subs}}=I_{0}{\left|\frac{r_{02}+r_{23}e^{-2i\varphi_{2}}}{1+r_{02}r_{23}e^{-2i\varphi_{2}}}\right|}^{2}$$

and

2 $$I_{\mathrm{memb}}=I_{0}{\left|\frac{r_{01}e^{i\left(\varphi_{1}+\varphi_{2}\right)}+r_{12}e^{-i\left(\varphi_{1}-\varphi_{2}\right)}+r_{23}e^{-i\left(\varphi_{1}+\varphi_{2}\right)}+r_{01}r_{12}r_{23}e^{i\left(\varphi_{1}-\varphi_{2}\right)}}{e^{i\left(\varphi_{1}+\varphi_{2}\right)}+r_{01}r_{12}e^{-i\left(\varphi_{1}-\varphi_{2}\right)}+r_{01}r_{23}e^{-i\left(\varphi_{1}+\varphi_{2}\right)}+r_{12}r_{23}e^{i\left(\varphi_{1}-\varphi_{2}\right)}}\right|}^{2}$$

where *r*_*ij*_ = (*ñ*_*i*_ – *ñ*_*j*_)/(*ñ*_*i*_ + *ñ*_*j*_) is the reflection coefficient at the interface between two media with wavelength-dependent complex refractive indices *ñ*_*i*_ and *ñ*_*j*_. The expression φ_*i*_ = 2π*ñ*_*i*_ *d*_*i*_/λ is the phase shift through a media as the light propagates through a medium of thickness *d*_*i*_ and complex refractive index *ñ*_*i*_. The different media are identified by numerical subscripts: 0 for air, 1 for the membrane, 2 for SiO_2_, and 3 for Si in our case. The wavelength (λ) ranges from 400 to 800 nm, which covers the visible light spectrum in the formulas. The intensity spectra from different surfaces are referred to as Airy’s formulas and can also be derived using the transfer matrix method (TMM) for a greater number of media if needed.^26^

### 2.5 Colorimetric Analysis

The evaluation involves a complicated procedure that necessitates three sequential stages to determine the red, green, blue (RGB) color codes of the membranes.^27–30^ First, the theoretical reflectance spectrum of membrane (*I*_memb_), mentioned previously, with theoretical light source, is converted to *XYZ* tristimulus values using the following integrals

3 $$\begin{array}{l} X=k\int R\left(\lambda \right)S\left(\lambda \right)x\left(\lambda \right)d\lambda \\ Y=k\int R\left(\lambda \right)S\left(\lambda \right)y\left(\lambda \right)d\lambda \\ Z=k\int R\left(\lambda \right)S\left(\lambda \right)z\left(\lambda \right)d\lambda \end{array}$$

where *R*(λ) is the reflectance spectrum and *S*(λ) is the normalized spectral power distribution of the theoretical light source (International Commission on Illumination, CIE, Illuminant A). *x*(λ), *y*(λ), and *z*(λ) are CIE 1931 standard observer—color matching functions. The color matching functions (CIE *xyz* 1931) and light source (CIE standard illuminant A) were sourced from the CIE database.^31,32^ The normalization constant *k* can be calculated using the expression *k* = 1/(∫*y*(λ)*S*(λ)­dλ).

Second, the *XYZ* tristimulus values are transformed into linear RGB utilizing the following formula

$$\begin{array}{c} \left[\begin{array}{l} R\\ G\\ B \end{array}\right]=M^{-1}[\begin{array}{l} X\\ Y\\ Z \end{array}],\\ M^{-1}=\left[\begin{array}{lll} 1.75625045 & -0.83309316 & -0.27019238\\ -1.17336058 & 2.2710351 & 0.05030631\\ 0.23849367 & -0.8744768 & 4.53137981 \end{array}\right] \end{array}$$ 4

where *M* is the transformation matrix, and it should be derived from the chromaticity coordinates of the sRGB primaries and the white point corresponding to CIE Illuminant A, accounting for the halogen lamp in the microscope.^33^

Lastly, we need to convert linear RGB color to standard sRGB color since γ correction considers the nonlinear response of human vision and typical display devices.^34^ This transformation can be executed using the following equation, where *C* represents one of the R, G, B channels derived from *XYZ* tristimulus values

$$\begin{array}{cc} 12.92\times C & if\;C\leq 0.031308\\ 1.05\times C^{1/2.4}-0.055 & if\;C>0.031308 \end{array}$$ 5

## 3 Results

### 3.1 Thickness Characterization via Atomic Force Microscopy

The target membranes on 290 nm SiO_2_/Si substrates were selected based on their colors, and their thicknesses were measured using AFM. A variety of colors were observed under optical microscopy as shown in Figure 1. The predominant colors identified include orange, green, red, and yellow, along with their different tones. Our findings indicate that bluish/greenish colors are notable in membranes only less than 200 nm, whereas membranes exceeding 700 nm tend to exhibit just a yellowish color. It should be noted that each membrane may exhibit uniform or varied thicknesses in a homogeneous or heterogeneous fashion. Consequently, membranes displaying color variations within a length range of approximately 5 μm were excluded from the analysis.

**Figure 1:**
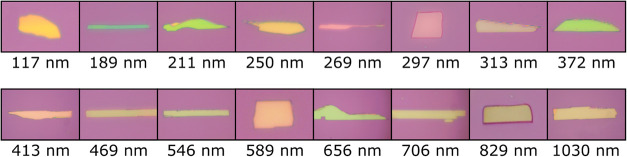
Optical images of (100) β-Ga_2_O_3_ membranes of different thicknesses with various colors on 290 nm SiO_2_/Si substrates. The thickness values, ranging from approximately 100 to 1000 nm, were precisely determined using atomic force microscopy and are annotated directly beneath each respective membrane in the image.

### 3.2 Optical Thickness Determination via Reflectance Contrast

Instead of AFM, the thickness of the membrane can be determined using an optical interference pattern. During the measurement of the reflectance spectrum, incident light from the microscope’s illumination source (Motic BA310MET-T) passes through the material and substrate. When incident light reaches the membrane, it reflects from the membrane’s upper surface as well as from each interface below it, such as the membrane-SiO_2_ and SiO_2_–Si interfaces. Every reflected beam follows a distinct optical trajectory, and they interfere with one another once they converge at the microscope, equipped with a CCD spectrometer (Thorlabs CCS200) to detect the reflectance spectra. The interference, whether constructive or destructive, is governed by Fresnel’s principles, where the parameters are the thicknesses and refractive indices of all the layers. If the wavelength-dependent complex refractive indices of all media (air, membrane, and substrate), given in Figure S2, and the thickness of the oxide layer of SiO_2_/Si are known, the thickness of the membrane can be found by fitting the theoretical and experimental optical contrast.^20–22^

Optical reflectance spectra from the substrate and membrane are needed to calculate optical contrast (*C*_e_). The acquisition time is adjusted to ensure that the intensity of the higher reflected surface, specifically from the membranes in our scenario, reaches a value of almost 1, thereby allowing for measurement normalization and reducing the effects of noise. This consistent value is also applied to the acquisition of another surface (substrate in this case). Figure 2a presents the reflectance spectra obtained for a 323 nm Ga_2_O_3_ membrane (red line) and a 290 nm SiO_2_/Si substrate (blue line) as an example. Subsequently, optical contrast can be determined using the formula below

6 $$C_{e}\left(\lambda \right)=\frac{I_{\mathrm{memb}}\left(\lambda \right)-I_{\mathrm{subs}}\left(\lambda \right)}{I_{\mathrm{memb}}\left(\lambda \right)+I_{\mathrm{subs}}\left(\lambda \right)}$$

where *I*_memb_ and *I*_subs_ are the intensities of reflection from the membrane and substrate, respectively. Theoretical reflectance spectra are needed to find theoretical contrast (*C*_t_) and were derived from Fresnel’s laws.

**Figure 2:**
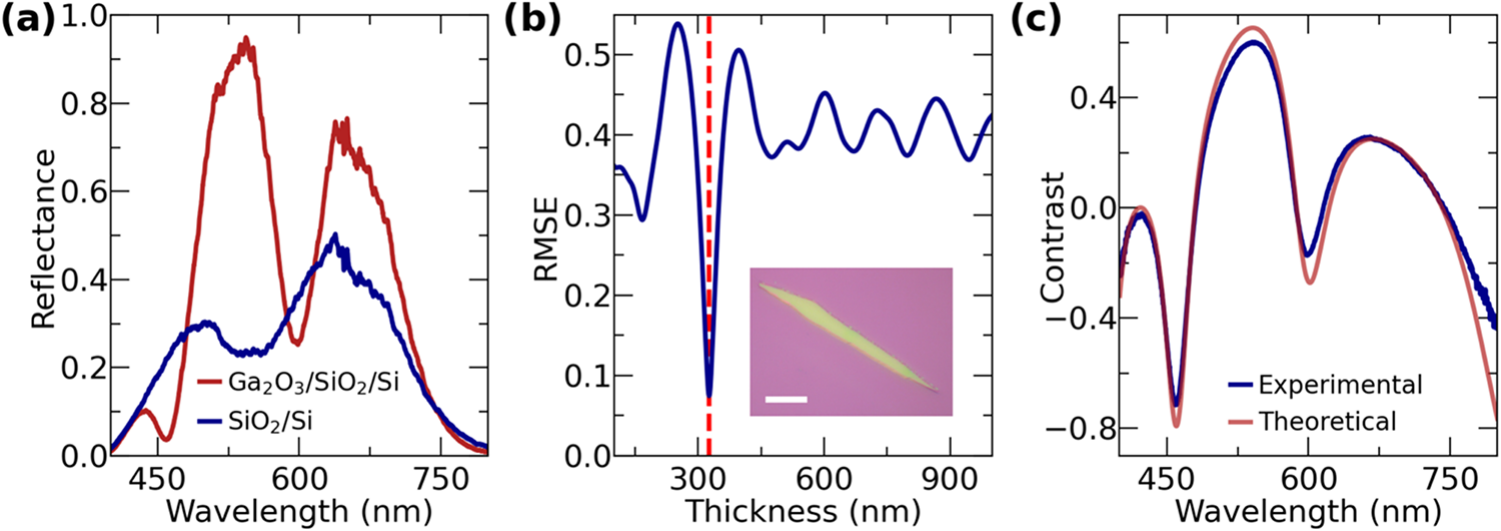
Thickness determination using optical interference pattern. (a) Reflectance spectra of 290 nm SiO_2_/Si substrate (blue line) and 323 nm (100) β-Ga_2_O_3_ membrane (red line) in the visible range. (b) Root mean square error (RMSE) result between the experimental optical contrast and the theoretical contrast calculated for various thicknesses in determining the thickness of a specific membrane. The vertical red dashed line indicates the position of minimum RMSE, which corresponds to the membrane thickness. The inset image shows the target membrane, with a scale bar representing 25 μm. (c) Comparison between the theoretical contrast (red line) at the thickness with the minimum RMSE and the experimental optical contrast of the membrane (blue line).

The thickness of the membrane can be found by comparing the theoretical contrast and optical contrast spectra. The root mean square error (RMSE) helps to do that, and the minimum RMSE gives the thickness of the membrane (*d*_1_ in this scenario), while other parameters are known in the formulas. Figure 2b presents RMSE results for a Ga_2_O_3_ membrane on a 290 nm thick SiO_2_/Si substrate. The analysis was performed by modeling the Ga_2_O_3_ thickness over a range from 100 to 1000 nm. The optical image of the sample is shown as an inset. The minimum RMSE value indicates an optimal film thickness of 326 nm for the Ga_2_O_3_ membrane. As illustrated in Figure 2c, the theoretical (red line) and optical contrast (blue line) spectra exhibit excellent agreement.

The accuracy of determining thickness from contrast is confirmed by comparing it to AFM findings. To achieve this, 50 diverse Ga_2_O_3_ membranes were chosen at random. The membranes’ thicknesses were assessed before employing AFM, and precise thickness measurements were obtained via AFM, as an example is provided in Figure S1. Figure 3 displays the findings with thicknesses determined by both AFM and the previously mentioned optical interference method. The angle of the best-fit line from the data points is 44.8 ± 0.5°, indicating a slight deviation from perfect alignment (45°). This outcome suggests that contrast can be employed for determining the thickness of submicron Ga_2_O_3_ membranes.

**Figure 3:**
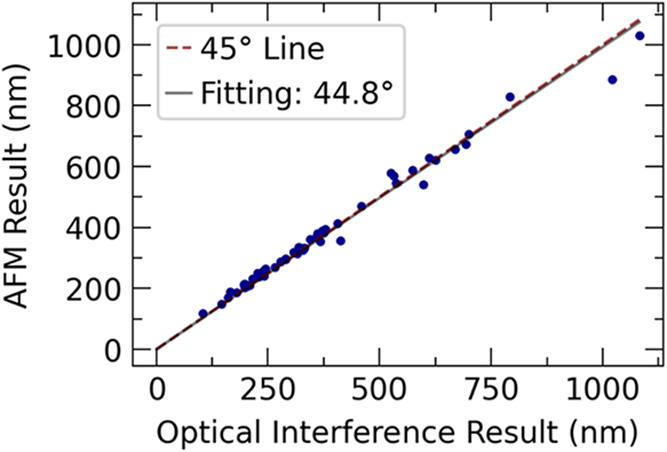
Comparison of thickness measurements obtained from AFM and optical interference for 50 Ga_2_O_3_ membranes with varying thicknesses. Scatter plot is AFM-measured thickness versus optical interference results (both in nm). The dashed red line represents the ideal 45° correspondence line, indicating perfect agreement between the two methods. The solid gray line shows the linear fit to the data, corresponding to a slope equivalent to 44.8 ± 0.5°, suggesting a slight systematic deviation from perfect correlation.

### 3.3 Colorimetric Analysis

While the exact thickness cannot be specified, the membrane’s color allows for an approximate estimation of its thickness range. Colorimetric analysis can be utilized to visualize the colors of membranes associated with varying thicknesses. Following colorimetric analysis procedures, the color of Ga_2_O_3_ membranes, assessed in 1 nm steps across a membrane thickness range from 0 to 1000 nm, was determined. Figure 4 presents the outcomes along with a color bar and optical images of various membranes. Comparison of colors between these images confirms the reliability of the colorimetric analysis, even though there is a small difference in the brightness. Moreover, the color bar exhibits periodic repetitions at approximately 150 nm intervals, with yellow tones becoming progressively more prominent. While red, green, and pink colors predominate in thinner membranes, orange and yellow colors become more dominant, particularly beyond the 600 nm threshold, as mentioned previously for experimental results. Consequently, the color bar offers an efficient and convenient method for the identification of the membranes.

**Figure 4:**
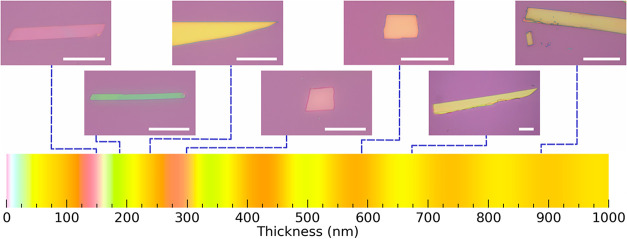
Appearance of (100) β-Ga_2_O_3_ membranes with thicknesses ranging from 0 to 1000 nm on 290 nm SiO_2_/Si substrates. The blue dashed lines connect optical images of the membranes to their corresponding thickness (determined by AFM) on the color bar to validate the colorimetric analysis. White scale bars in the optical images are 25 μm.

The color variation in Ga_2_O_3_ on 290 nm SiO_2_/Si has been discussed up to this point in the article due to its frequent use in the literature. Nevertheless, the 290 nm oxide film in the substrate may not be optimal for achieving a distinguishable contrast across different membranes to identify them easily. Consequently, total color difference (TCD) analysis was conducted as a function of both the Ga_2_O_3_ film thickness (0–1000 nm) and the SiO_2_ layer thickness (0–500 nm) on a Si substrate. TCD analysis details are given in the Supporting Information. Figure 5a illustrates the result of the analysis in a color map graph. If the TCD value increases, it indicates that the substrate and membrane are positioned farther apart from each other in the color space. Therefore, thinner membranes are more visible on approximately 220 nm SiO_2_/Si; in contrast, thicker membranes on substrates with a thinner oxide layer are harder to distinguish.

**Figure 5:**
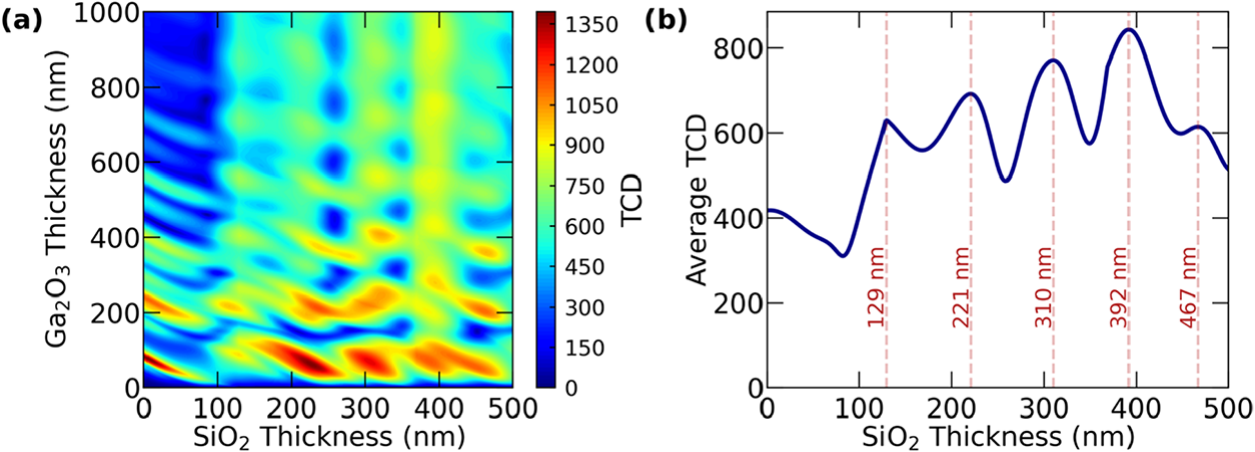
Analysis of total color differences (TCD) for (100) β-Ga_2_O_3_ membranes at various thicknesses on SiO_2_/Si substrates with varying thicknesses of the oxide layer. (a) TCD color map illustrating all possible outcomes. The vertical axis indicates the thickness of Ga_2_O_3_, specifying the thickness value used in the calculation of TCD value. The horizontal axis shows the SiO_2_ thickness used in the calculation. TCD values are depicted through a color bar positioned next to the principal color map. (b) The mean TCD across each Ga_2_O_3_ thickness was assessed by using TCD data of color map in (a). To determine the ideal SiO_2_ thickness for optimal membrane visibility, an average across the membrane’s thickness was computed, revealing that superior membrane visibility is achieved with SiO_2_/Si substrates measuring 129, 221, 310, 392, and 467 nm in thickness.

However, the color map does not give a complete idea about each thickness value of the oxide layer. Therefore, TCD averages across the membrane’s thickness were calculated as shown in Figure 5b. The local maximum of TCD indicates that the substrate with 129, 221, 310, 392, and 467 nm thick oxide layers gives a better contrast than other thicknesses. In the average TCD, the thickness of Ga_2_O_3_ spans from 0 to 1000 nm, but different local maxima emerge when considering a narrower thickness range, notably for thinner cases, as given in Figures S3 and S4. It is noteworthy that the positions of the local maxima in TCD values were determined to be approximately identical with different ranges of Ga_2_O_3_ thicknesses.

After TCD analysis, the appearance of Ga_2_O_3_ membranes was computed at SiO_2_ thickness values, indicating the local maximum value of TCD. The results are listed in Figure 6. Ga_2_O_3_ membranes of different thicknesses exhibit a similar color trend on oxide layers of different thicknesses as membranes on a 290 nm SiO_2_/Si substrate. Although colors become more noticeable on oxide with reduced thickness, the color trend at greater oxide thicknesses primarily shifts toward a yellowish color. As a result, the thickness of Ga_2_O_3_ membranes can be more easily identified on a SiO_2_/Si substrate with a thinner oxide layer than with a thicker one. As an example, Figure S5 shows the results for membranes on bare silicon, showing good agreement between the color bar and the actual colors.

**Figure 6:**
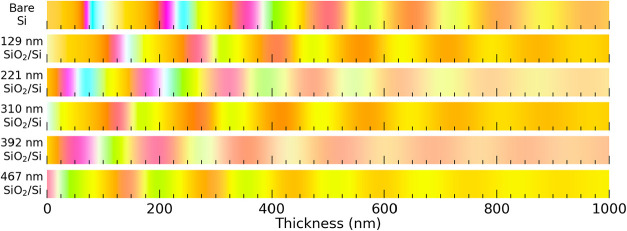
Appearance of Ga_2_O_3_ membranes with thicknesses ranging from 0 to 1000 nm. The oxide layer thicknesses of SiO_2_/Si substrates vary as follows: 0, 129, 221, 310, 392, and 467 nm, with all of them except the bare silicon exhibiting a higher average TCD.

## 4 Conclusions

Optical interference has been demonstrated to be a viable tool to determine the thickness of Ga_2_O_3_ membranes in a nondestructive and efficient manner, fusing Fresnel’s law, as shown here for (100)-oriented β-Ga_2_O_3_. Atomic force microscopy results confirm the precision of this method by comparing thickness measurements obtained through these two distinct approaches.

The optical interference causes different color appearances of membranes with various thicknesses, depending on the substrate. The thickness dependency of membrane color was demonstrated by colorimetric analysis on a 290 nm SiO_2_/Si substrate. In this way, the color bar of the Ga_2_O_3_ generated by colorimetry can facilitate the identification of the submicrometer membrane thicknesses. By integrating these techniques, the initial stage of the research on this kind of Ga_2_O_3_ structure before device fabrication can be accelerated via noncontact, user-friendly, high-throughput thickness characterization.

Although a 290 nm SiO_2_/Si substrate was used in the experiments described in this paper, other silicon oxide thicknesses may provide an improved optical contrast. Accordingly, TCD analysis was employed to determine the SiO_2_ layer thicknesses on Si substrates that delivered optimal contrast for Ga_2_O_3_ membranes, facilitating their visual identification under an optical microscope. These findings serve as practical guidelines in Ga_2_O_3_ research for selecting appropriate substrate oxide thicknesses.

## Supplementary Material



## Data Availability

The data generated in this study and the custom python scripts used for data analysis and the generation of figures have been deposited in the Zenodo database (https://doi.org/10.5281/zenodo.18236175).

## Notes

Overview of CIE standardization systems and the mathematical procedures for converting between linear RGB and sRGB color spaces; wavelength-dependent complex refractive index spectra for (100) β-Ga_2_O_3_, SiO_2_, Si, and air; mathematical derivation of Total Color Difference (TCD) using *La***b** coordinates; additional TCD visibility results for narrow β-Ga_2_O_3_ thickness ranges (0–400 and 0–200 nm); and experimental validation of colorimetric results on bare silicon substrates (PDF)
