# Supplementary material for: Determination of the Thickness of Nanometer-Thick β‑Ga2O3 Membranes from Optical Interference and Colorimetric Analysis for Applications in Next-Generation Semiconductors
Source: ACS Appl Nano Mater. 2026 Jun 27;9(27):13126–32. doi: 10.1021/acsanm.6c02052 (PMC13366758; doi:10.1021/acsanm.6c02052)
Supplement: Supplementary file 1 [file an6c02052_si_001.pdf]

Supporting Information

Determination of Thickness of Nanometer-Thick β-Ga_2O_3 Membranes from Optical Interference and Colorimetric Analysis for Applications in Next Generation Semiconductors

Onur Çakıroğlu*, Paula Pérez-Peinado, Emilio Nogale, Bianchi Méndez

Departamento de Física de Materiales, Facultad de Ciencias Físicas,
Universidad Complutense de Madrid, 28040 Madrid, Spain
** Corresponding autor: ocakirog@ucm.es*

1. Measurements and Data Analysis in Atomic Force Microscopy

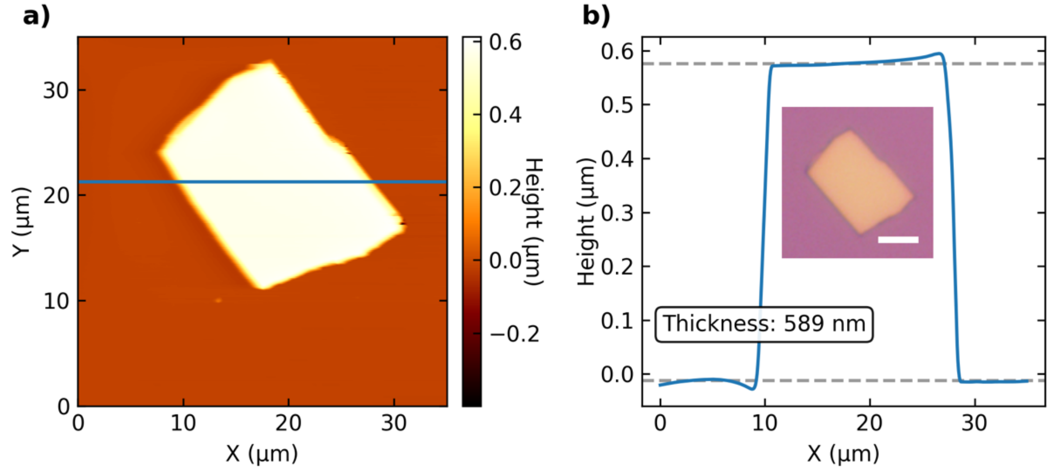

Figure S1: *Thickness characterization by atomic force microscopy (AFM).* ***a)*** *AFM topographical image of one of the membranes investigated in this study. The horizontal and vertical axes denote the lateral dimensions of the scan area, while the color bar indicates the measured height. The blue lines indicate the locations of the height profiles.* ***b)*** *Thickness profile obtained by averaging seven adjacent scan lines centered on the blue lines shown in a). The inset shows an optical image of the same membrane. The scale bar corresponds to 5 µm.*

2. CIE Standardization[1]

The CIE (Commission Internationale de l'Eclairage) is a global organization tasked with establishing standards and standardized measurement procedures in illuminating engineering and closely related areas. The organization created the standardization of colorimetry too. Three main systems (CIE 1931, CIE 1964 and CIE 1976) for the colorimetry were defined with different purposes. The differences between them are as follows:

- **CIE 1931:** It is the initial attempt for applied colorimetry and color-matching functions (x,y,z) was developed considering ideal observer with 1-4° angular subtense which is the size of the visual field. This area is more or less the same size as the most active receptor region (the foveal region) of the eyes.
- **CIE 1964:** Although the foveal region is mostly dominated in view, other regions of the retina are also responsible. Therefore, the system was extended to 10° angular subtense.
- **CIE 1976:** Ideal observers detect the light intensity linearly like sensors and digital cameras. However, real human eyes don't pretend in the same fashion. For instance, light that is double in brightness doesn't appear twice as bright to human perception. Consequently, the correction was considered.

These systems determine the main mechanism in the conversion of reflection into RGB color code as shown later. Nonetheless, CIE 1931 was selected for the conversion process, as it sufficiently enables color matching in optical images.

3. Linear RGB and sRGB Representations and Their Conversions[1–3]

A "Linear RGB" system is characterized by Red, Green, and Blue (**RGB**) component values that are linearly proportional to light intensity. The range for each channel extends from 0 to 1 (or sometimes from −0.5 to 0.5). Nevertheless, the human visual system does not perceive each color with the same sensitivity; it exhibits greater sensitivity to darker colors compared to brighter ones. Therefore, sRGB, an abbreviation for standard RGB, is employed by monitors as well as JPEG and PNG image formats. The sRGB range for each channel extends from 0 to 255 (or 8 bits).

The adjustment of contrast and brightness can be achieved through the application of Linear RGB. Consequently, it is necessary to convert sRGB color to Linear RGB to perform this operation. Conversion from sRGB to Linear RGB can be found by this formula:

$$c' = \begin{cases} c\,/\,12.92 & if\ c \leq 0.04045 \\ [(c + 0.055)/1.055]^{2.4} & if\ c > 0.04045 \end{cases} \tag{1}$$

where c denotes a channel code of the RGB color values in Linear RGB space while c'represent sRGB space. Reverse conversion (from Linear RGB to sRGB) can be performed by the following formula;

$$c = \begin{cases} c' \cdot 12.92 & if\ c' \leq 0.0031308 \\ 1.055\left(c'^{\ 1/2.4} - 0.055\right) & if\ c' > 0.0031308 \end{cases} \quad (2)$$

4. Complex Refractive Index of Air, Ga_2O_3, SiO_2 and Si

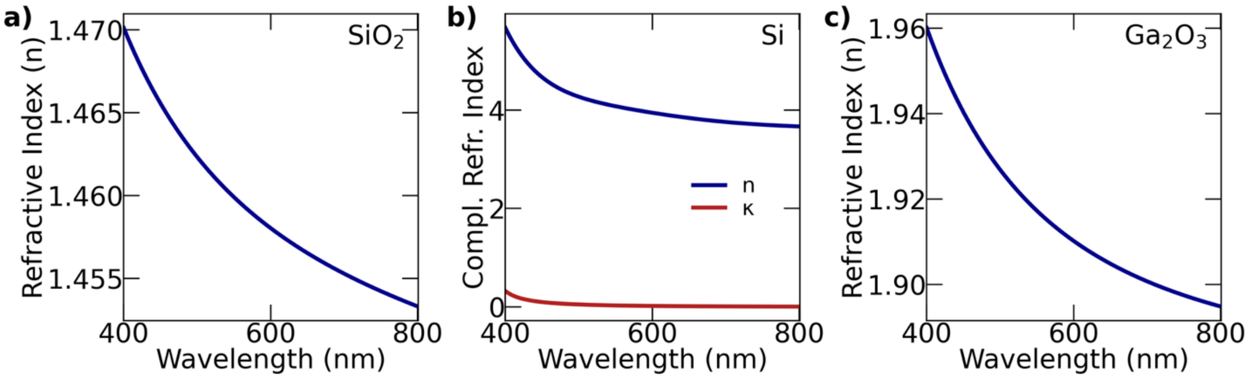

Figure S2: *Spectra of (complex) refractive index of SiO_2, Si and (100) β-Ga_2O_3.[4–6] Both SiO_2 and Ga_2O_3 are considered transparent, which means their extinction coefficient (κ) equals zero. These values are used for theoretical contrast computation, colorimetric analysis, and TCD calculations, considering a wavelength range from 400 to 800 nm.*

The complex refractive index of air has only a real component that was considered to be 1 through all wavelength range in this study. The refractive index for Si, depicted in Figure 1, was used in its complex forms, while SiO_2 and (100) β-Ga_2O_3 are considered transparent, indicating they lack a complex component and consist only of real parts. It should be noted that for membranes aligned along any crystallographic direction other than (100), the only change required is to adapt the complex refractive index associated with that particular orientation.

5. TCD Analysis to See Better Visibility on SiO_2/Si[1]

The Total Color Differences (TCD) value can be associated with the visibility of a membrane on a substrate. Briefly, XYZ tristimulus coordinates of the membrane and substrate should be determined until RGB values by the method discussed in **Section 3**. However, these values of the membranes aren't enough to calculate TCD although they are acceptable to calculate RGB values of the membranes.

The XYZ space was converted to La^*b^* space via these conditions:

$$
\begin{aligned}
L &= \begin{cases} 116(Y/Y_0)^{1/3} - 16 & if \quad Y_r > \epsilon \\ 903.3(Y/Y_0) & \quad Y_r \leq \epsilon \end{cases} \\
a^* &= \begin{cases} 500\left[(X/X_0)^{1/3} - (Y/Y_0)^{1/3}\right] & if \quad X_r > \epsilon \\ 3893.5\,[X/X_0 - Y/Y_0] & \quad X_r \leq \epsilon \end{cases} \\
b^* &= \begin{cases} 200\left[(Y/Y_0)^{1/3} - (Z/Z_0)^{1/3}\right] & if \quad Z_r > \epsilon \\ 1557.4\,[Y/Y_0 - Z/Z_0] & \quad Z_r \leq \epsilon \end{cases}
\end{aligned}
\tag{3}
$$

where $X_r = X/X_0$, $Y_r = Y/Y_0$ and $Z_r = Z/Z_0$ while X_0, Y_0 and Z_0 are 1.09850, 1.0000, 0.35585 respectively.[7] These values are the white source references of tristimulus components for halogen light source and consequently different light sources possess varying values. The value of ϵ is 0.008856. After La^*b^* coordinates must be calculated for membrane and substrate separately, distance between these two points gives the TCD values by this formula

$$TCD = [(\Delta L)^2 + (\Delta a^*)^2 + (\Delta b^*)^2]^{1/2} \tag{4}$$

In which ΔL, Δa^* and Δb^* denote the subtraction La^*b^* values of the membrane from ones of the substrate. The process is iterated for every thickness value of the Ga_2O_3 membrane and the oxide layer of the SiO_2/Si substrate.

6. TCD Results in a Narrower Thickness Range of β-Ga_2O_3 Membranes

Even if TCD values on color map do not change, TCD average in the narrower range of β-Ga_2O_3 thickness result in different outcomes. Nevertheless, there is limited discussion regarding the variations in local maxima of the TCD average (or the optimal visibilities for β-Ga_2O_3 membranes) as illustrated in the Figures below. Therefore, a wider range in the primary manuscript does not negatively affect membrane visibility.

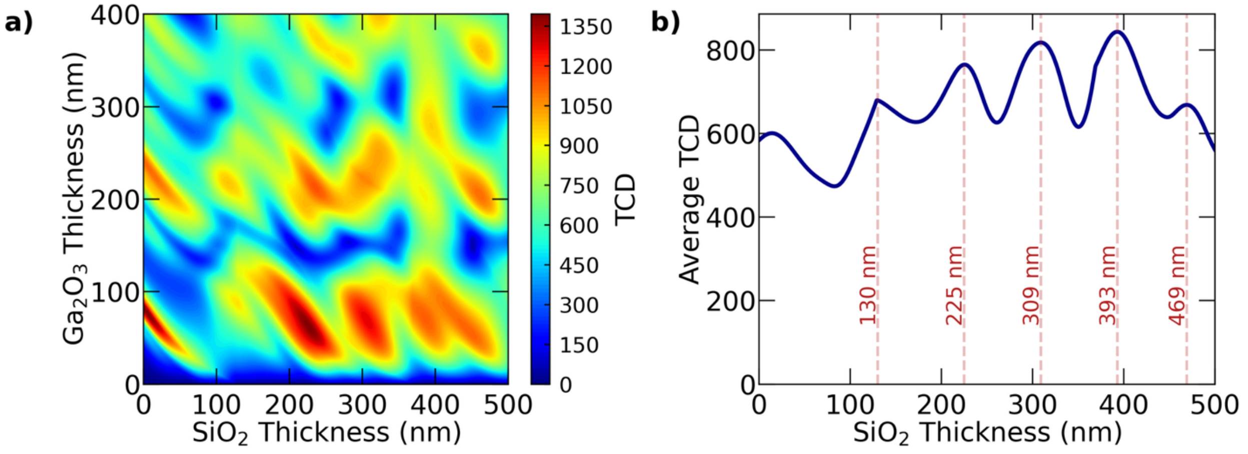

Figure S3: *TCD analysis for narrower range (0-400nm) of (100) β-Ga_2O_3 thicknesses.* ***a)*** *TCD color map illustrating all possible outcomes.* ***b)*** *The mean TCD across each Ga_2O_3 thickness was assessed by using TCD data of color map.*

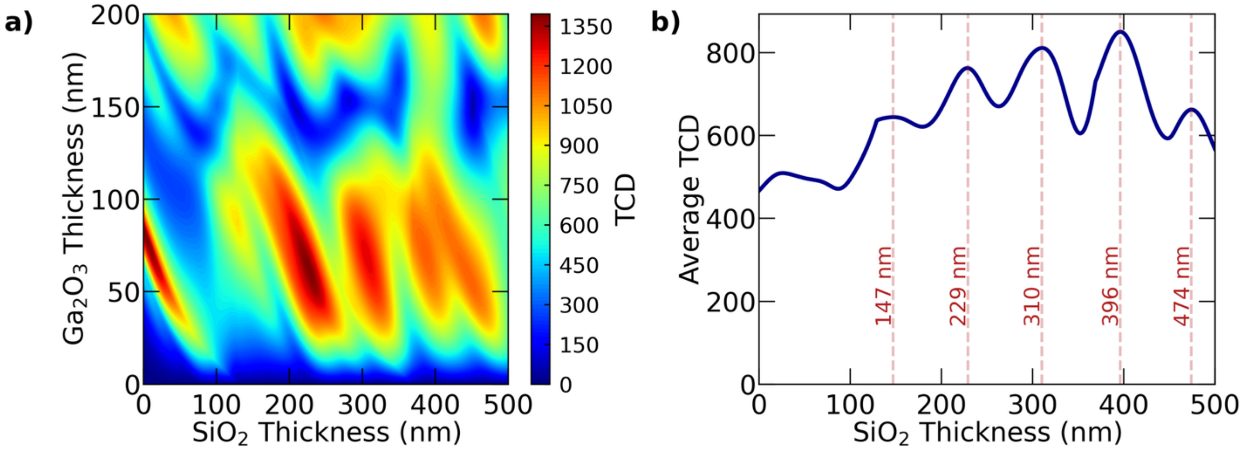

Figure S4: *TCD analysis for narrowest range (0-200nm) of (100) β-Ga_2O_3 thicknesses.* ***a)*** *TCD color map illustrating all possible outcomes.* ***b)*** *The mean TCD across each Ga_2O_3 thickness was assessed by using TCD data of color map.*

7. Comparing Experimental Appearance of (100) β-Ga_2O_3 on Bare Silicon with Colorimetric Results

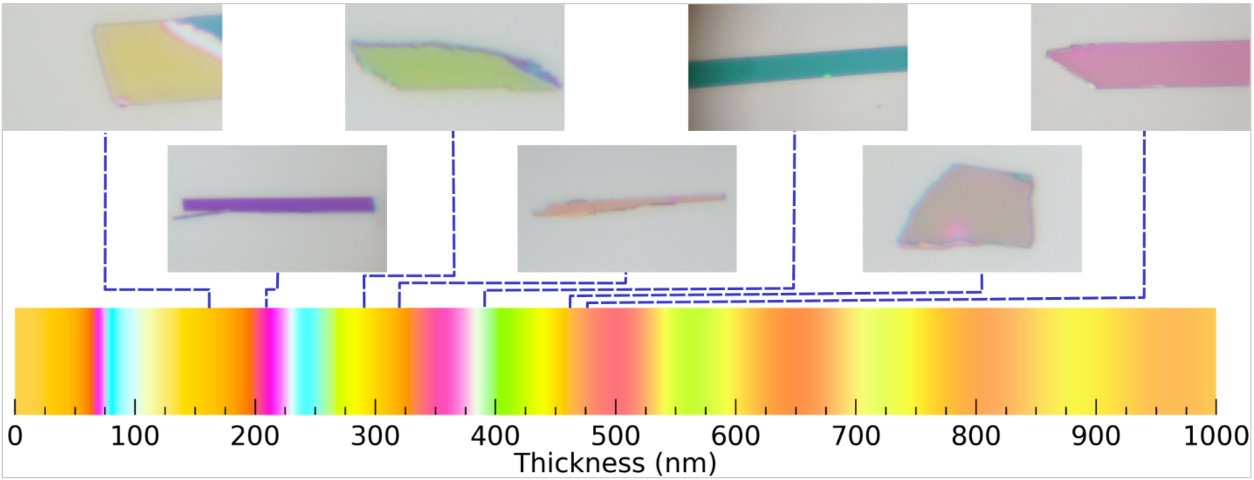

Figure S5: *Appearance of (100) oriented β-Ga_2O_3 membranes with thicknesses ranging from 0 nm to 1000 nm on bare Si substrates. The blue dashed lines connect optical images of Ga_2O_3 membranes to their corresponding thickness (determined by the optical interference analysis) on the color bar to validate.*

8. Color Repetition over Thickness

The thin-film interference criterion for a complete color shift with changing thickness is given by $\Delta d \approx \lambda/2n$, where d, λ, and n denote the membrane thickness, the dominant wavelength of the light source, and the average refractive index of the membrane, respectively. Taking λ = 600 nm for a halogen lamp and n ≈ 1.93 for β-Ga_2O_3 gives $\Delta d \approx$ 155 nm. Consequently, a repetition of the

observed colors in the color charts is expected at intervals of about 155 nm in thickness theoretically.

However, this means that color and thickness are no longer in a one-to-one relationship, so determining the thickness requires either the Fresnel-based method shown here or AFM. Color charts indicate that the color shifts to orange at higher thickness values, while lower thickness values yield brighter colors. Thus, these charts can be used to quickly identify membranes under an optical microscope, especially for thin membranes.

At greater membrane thicknesses, the orange color is attributed to the halogen light source, since its dominant wavelength lies in the yellow-orange-red range. As the membranes thicken, their color is determined by the light source rather than by optical interference.

9. Error Margins in Thickness Determination via Optical Interference

Light path defects, variations in focus, contamination at interfaces, or microscopic irregularities of the membrane surface can experimentally introduce errors in determined thickness.

Moreover, uncertainties in refractive index of SiO_2 and Ga_2O_3, and in thickness of SiO_2 layer can computationally cause errors. Although Fresnel equation is non-linear, their effects can be shown on 323 nm thick Ga_2O_3 as a case study. A 1% change in n_{sio2}, n_{Ga2O3} and d_{sio2} causes %0.65, %0.99 and %0.63 errors in thickness determination and overall uncertainty becomes ± 5.2 nm or %1.6. The impact of changes in the refractive index of the silicon layer is limited because a semi-infinite approximation is used in the Fresnel-based calculation. As a result, even a 10% change in the silicon refractive index leads to only about a 0.03% variation in the estimated thickness of the β-Ga_2O_3 layer.

If the margin of error were computed in the AFM comparison, an average error of about 0.7% would be obtained across all membranes measured in this study. Therefore, the method exhibits an acceptable error margin for β-Ga_2O_3.

References

(1) Wyszecki, G.; Stiles, W. S. *Color Science: Concepts and Methods, Quantitative Data and Formulae*, 2. ed., Wiley classics library ed.; Wiley classics library; Wiley: New York, NY Weinheim, 2000.

(2) *Matlab-Understanding Color Spaces and Color Space Conversion*. https://www.mathworks.com/help/images/understanding-color-spaces-and-color-space-conversion.html (accessed 2025-11-19).

(3) *Gitlab - GNOME Gegl Colors*. https://gitlab.gnome.org/GNOME/gegl/-/blob/master/opencl/colors.cl (accessed 2025-11-19).

(4) Malitson, I. H. Interspecimen Comparison of the Refractive Index of Fused Silica*,†. *J. Opt. Soc. Am.* **1965**, *55* (10), 1205. https://doi.org/10.1364/JOSA.55.001205.

(5) Schinke, C.; Christian Peest, P.; Schmidt, J.; Brendel, R.; Bothe, K.; Vogt, M. R.; Kröger, I.; Winter, S.; Schirmacher, A.; Lim, S.; Nguyen, H. T.; MacDonald, D. Uncertainty Analysis for the Coefficient of Band-to-Band Absorption of Crystalline Silicon. *AIP Advances* **2015**, *5* (6), 067168. https://doi.org/10.1063/1.4923379.

(6) Carrasco, D.; Nieto-Pinero, E.; Alonso-Orts, M.; Serna, R.; San Juan, J. M.; Nó, M. L.; Jesenovec, J.; McCloy, J. S.; Nogales, E.; Méndez, B. Temperature-Dependent Anisotropic Refractive Index in β-Ga2O3: Application in Interferometric Thermometers. *Nanomaterials* **2023**, *13* (6), 1126. https://doi.org/10.3390/nano13061126.
(7) Lindbloom, B. *Useful Color Equations*. http://brucelindbloom.com/index.html?Eqn_RGB_XYZ_Matrix.html (accessed 2025-11-19).
